# Comparison of Knowledge, Attitude, and Practice of Nursing and Medical Students in Kermanshah, Iran, about Toothbrush Maintenance and Use

**DOI:** 10.1155/2021/6669029

**Published:** 2021-06-11

**Authors:** Maryam Janatolmakan, Saber Kakazadeh, Bahare Andayeshgar, Faranak Jafari, Alireza Khatony

**Affiliations:** ^1^Clinical Research Development Center, Imam Reza Hospital, Kermanshah University of Medical Sciences, Kermanshah, Iran; ^2^Student Research Committee, Kermanshah University of Medical Sciences, Kermanshah, Iran; ^3^School of Health, Kermanshah University of Medical Sciences, Kermanshah, Iran; ^4^Social Development and Health Promotion Research Center, Health Institute, Kermanshah University of Medical Sciences, Kermanshah, Iran; ^5^Infectious Diseases Research Center, Kermanshah University of Medical Sciences, Kermanshah, Iran

## Abstract

**Background:**

To promote oral health in society, medical and nursing students are required to have acceptable knowledge, attitude, and practice with respect to correct maintenance and use of a toothbrush. This study explored the knowledge, attitude, and practice of Iranian medical and nursing students about the correct maintenance and use of a toothbrush.

**Methods:**

A total of 260 nursing students and 320 medical students were randomly recruited. Data were collected by a researcher-made questionnaire on the knowledge, attitude, and practice about toothbrush maintenance and use. Data were analyzed using descriptive and inferential statistics.

**Results:**

The mean scores of knowledge, attitude, and practice were 54.53 ± 17.18, 49.67 ± 19.17, and 19 ± 51.50 in the nursing students and 54.17 ± 21.42, 51.18 ± 87.28, and 49.87 ± 17.52 out of 100 in the medical students, respectively. There was no significant difference between the medical and nursing students in the mean score of knowledge. The medical and nursing students had a similar attitude and practice in most items of toothbrush maintenance and use.

**Conclusion:**

The medical and nursing students had average knowledge, attitude, and practice regarding toothbrush maintenance and use, which is not acceptable considering their job nature. Hence, interventional measures should be taken to enhance their knowledge, attitude, and practice.

## 1. Introduction

Oral health affects different aspects of life, including physical, mental, and social health [1, 2]. Tooth brushing is the most basic and common means of maintaining oral health [3]. Tooth brushing eliminates dental plaques, food residues, and stains on the tooth surface [4, 5]. Despite the efficacy of toothbrushes and their role in oral health, incorrect use and maintenance of toothbrushes are dangerous, which is followed by risks such as oral infections [6, 7]. Knowledge of correct maintenance and use of toothbrushes plays a pivotal role in oral health [8]. Washing the toothbrush before and after use, replacing the toothbrush every 3–4 months, and regular tooth brushing twice a day for 2–3 minutes are some strategies for the maintenance and use of toothbrushes [8–10]. Nowadays, nurses have the most important role in the health education domain [11]. On the other hand, education in medical and nursing disciplines will be practically useless unless it brings about useful changes in the personal health of these students [12]. In this regard, it is highly important that the medical and nursing students, the main healthcare providers, have adequate knowledge about correct maintenance and use of toothbrushes because they are at the center of public interest and are considered role models [13]. Hence, if they have poor knowledge, attitude, and practice, the patients will get the main damage [12]. A study in Kuwait (2003) showed that medical students had little knowledge of oral health [14]. Another study in Hong Kong (2017) reported that nursing students had poor knowledge about oral health, with no significant relationship between their knowledge and practice [15]. As for the effect of education on the knowledge, attitude, and practice of students, a study on Bangladeshi students (2016) showed that teaching the oral health principles promoted their knowledge, attitude, and practice [16]. A study in Iran (2010) on oral health indicated no significant difference between the medical and nonmedical students in terms of the frequency of tooth brushing, but dental flossing was more frequent among the medical students [17].

Since knowledge about the principles of oral health, especially correct use of tooth brushing in medical students, is highly important and that no study has ever investigated the knowledge, attitude, and practice of nursing and medical students about toothbrush maintenance and use, the present study was designed to compare the knowledge, attitude, and practice of nursing and medical students about toothbrush maintenance and use. The results of this study can be helpful to the health authorities and policymakers in adopting necessary interventional measures.

## 2. Materials and Methods

### 2.1. Study Design

This descriptive-analytical study was performed on the medical and nursing students from February to March 2019. The study was performed according to STROBE instructions.

### 2.2. Study Question

In this study, we sought to answer the following question: “What levels of knowledge, attitude, and practice do medical and nursing students have about toothbrush maintenance and use?”

### 2.3. Study Hypothesis

The medical and nursing students have different levels of knowledge, attitude, and practice about toothbrush maintenance and use.

### 2.4. Sample and Sampling Method

The study population comprised all medical and nursing students studying in the second semester of the academic year 2018–2019. Since the study population included 785 and 1859 nursing and medical students, respectively, the sample size was estimated to be 260 nursing and 320 medical students using the following Cochrane's formula with 95% confidence level, the first type error equal to 5%, and *p*=0.5%:

(1) $$\begin{array}{c} n=\frac{N\times P\left(1-P\right)\times Z_{1\left(\alpha /2\right)}^{2}}{\left(N-1\right)\times d^{2}+P\left(1-P\right)\times Z_{1\left(\alpha /2\right)}^{2}}. \end{array}$$

The samples were selected by the random sampling method. For this purpose, the list of students' names was taken from the education department and numbered. The samples were then selected using a table of random numbers. In the next step, the researcher referred to the samples according to the class schedule and in case of consent to participate in the study, a questionnaire was provided to them. If the person did not want to participate in the study, the person before or after him/her would be replaced according to the list of names. The inclusion criteria consisted of study in the second semester of the 2019–2020 academic year, consent to participate in the study, and studying in the medical and nursing discipline. Incomplete completion of the questionnaires was the exclusion criterion.

### 2.5. Measurement Instruments

Data were collected by a researcher-made questionnaire adopted from the previous studies [12, 16, 18]. Content validity method was used to analyze the validity of the questionnaire, which included two methods: qualitative and quantitative. In the qualitative part, the questionnaire was given to 12 medical, dental, and nursing faculty members, and their corrective comments were applied. In the qualitative part, based on the opinions of experts, the content validity indexes (CVI) were calculated for the scales of knowledge, attitude, and practice, which were equal to 0.87, 0.89, and 0.88, respectively. The reliability of the questionnaire was confirmed by the test-retest method. To this end, the questionnaire was first given to 30 medical and nursing students to complete. After two weeks, the same questionnaire was given to them again to complete. The correlation coefficients for the scales of knowledge, attitude, and practice were 0.87, 0.88, and 0.86, respectively.

The questionnaire is comprised of four sections. The first section was about the demographic information of the participants, including age, gender, and major. The second section consisted of 10 items on the students' knowledge about toothbrush maintenance and use. Some items of this section included “How often do you brush your teeth in a day?”, “What is a proper place for keeping a toothbrush?”, and “How much time is good for tooth brushing?”. To calculate the total score, the correct and false responses were given scores 1 and 0, respectively. The score range of knowledge was multiplied by 100 and classified as poor knowledge (≤49), average knowledge (50–74), and good knowledge (≥75).

The third section included 6 two-option items, including agree and disagree, on the students' attitude toward toothbrush maintenance and use. Some items of this section were “The toothbrush material affects its life,” “Rinsing the toothbrush is enough to reduce its contamination,” and “It is usually better to keep the toothbrush in water closet.” To compute the total score of attitude, with the exception of items 1 and 4, the opposing answers were given a score of 1 and those who agreed were given a score of 0. The score range of attitude was multiplied by 100 and classified as poor attitude (≤49), average attitude (50–74), and good attitude (≥75).

The fourth section consisted of 10 items on the students' practice in toothbrush maintenance and use. Some items of this section included “What method do you use for tooth brushing?”, “How do you wash your toothbrush?”, and “How long do you brush your teeth?” To calculate the total score, the correct and false responses were given scores 1 and 0, respectively. The range of scores was multiplied by 100 and classified as poor practice (≤49), average practice (50–74), and good practice (≥75).

### 2.6. Data Gathering

Having taken the approval of the ethics committee of the university, the researcher referred to the education departments of the nursing and medical faculties, obtained and coded the students' names, and selected the samples by systematic random sampling. For this purpose, the researcher referred to the students during the breaks between classes and explained the research objectives to them. If they were willing to participate in the study, they were given the questionnaire to complete. To increase the reliability of the data in this study, the questionnaires were anonymous, the participants were visited in proper time, the questionnaires were given to the participants at an appropriate time, and questionnaires with more than 20% missing data were excluded from the study.

### 2.7. Data Analysis

Data were analyzed by the Statistical Package for Social Sciences (SPSS-16 Inc., Chicago, IL, USA) using the descriptive (mean, standard deviation, median, interquartile range, and percentage) and inferential statistics (Kolmogorov–Smirnov test, Chi-square test, and Mann–Whitney *U* test). Kolmogorov–Smirnov test was used to determine the distribution of age and total scores of knowledge, attitude, and practice, which indicated the abnormal distribution of the given variables. Further, the chi-square test was used to compare the frequency of the medical and nursing students' responses to the items of different sections of the questionnaire and the percentage of gender in both groups. Mann–Whitney *U* test was also used to compare age and the scores of knowledge, attitude, and practice in the medical and nursing students. The significance level for all tests was less than 0.05.

### 2.8. Ethical Considerations

The present study was approved by the Ethics Committee of KUMS, with the reference no. IR.KUMS.REC.1397.798. All participants were assured their demographic information would be kept confidential. Informed written consent was taken from all of the participants.

## 3. Results

The study sample included a total of 580 students, 260 nursing students, and 380 medical students. Most of the nursing students (*n* = 142, 54.6%) were female and most of the medical students (*n* = 170, 53.1%) were male. The medical and nursing students were homogenous in terms of gender. The results of the KS test showed an abnormal distribution of age (*p* < 0.001), so the median and interquartile range (IQR) were used to compare the medical and nursing students concerning age. The median and IRR of age in the nursing and medical students were 21 and 3, respectively, indicating no statistically significant difference between them (Table 1).

The median and IQR of the total score of medical and nursing students on toothbrush maintenance and use were equal, 60 (out of 100) and 30, respectively. The results of the Mann–Whitney *U* test showed no statistically significant difference between the medical and nursing students regarding toothbrush maintenance and use (Table 2). The frequency and percentage of responses to different questions of knowledge section of the questionnaire were compared between the medical and nursing students by Chi-square test, which indicated no significant difference between them. For instance, 47.5% of medical students and 53.1% of nursing students selected the “three times” option of the question “How often do you brush your teeth in a day?”. Further, 47.5% of medical students and 55% of nursing students selected the option “2–3 minutes” in response to the item “How long do you brush your teeth?”. Moreover, 61.9% of medical students and 67.7% of nursing students chose the option “vertical-rotary” option in response to the item “What is the best tooth brushing technique?” (Table 3).

Regarding the attitude of the medical and nursing students toward toothbrush maintenance and use, the findings showed the median and IQR of each group were 50 (out of 100) and 33.33, respectively. The results of the Mann–Whitney *U* test indicated no significant difference between the medical and nursing students' total scores of attitude toward toothbrush maintenance and use (Table 2). The comparison of the frequency of responses to different questions of attitude section showed the medical and nursing students had a similar attitude toward toothbrush maintenance and use in all items except the effect of material on the life of toothbrush (*p*=0.012). Moreover, 91.5% of nursing students (238 out of 260 students) and 84.7% of medical students (271 out of 320 students) agreed on the effect of material on the life of toothbrushes (Table 4).

As for the practice of the nursing and medical students in toothbrush maintenance and use, the findings showed the median and IQR of 50 (out of 100) and 30, respectively. The mean total scores of the medical and nursing students' practice in toothbrush maintenance and use indicated no significant difference between them (Table 2). The results indicated the nursing and medical students had similar practice in all items except for the time of using toothbrush cap (*p*=0.02) so that 58.5% of nursing students (152 out of 260 students) used toothbrush cap after the toothbrush dried, but 51.2% of medical students (164 out of 320 students) used toothbrush cap immediately after washing the toothbrush. Most of the medical and nursing students (64.4% vs. 63.1%) used medium bristle toothbrushes and kept their toothbrushes out of the bathroom (70.6% vs. 68.8%). Further, 58.1% of medical students and 59.6% of nursing students used the vertical-rotary tooth brushing method (Table 5).

## 4. Discussion

The present study compared the knowledge, attitude, and practice of nursing and medical students about toothbrush maintenance and use. To promote the oral health of people in society, medical and nursing students are required to have acceptable knowledge, attitude, and practice with respect to toothbrush maintenance and use.

The findings showed the medical and nursing students had similar and average knowledge, attitude, and practice regarding toothbrush maintenance and use, which is not acceptable considering their educational role. On the other hand, these students have important responsibilities such as providing personal health principles to society, including oral health. Hence, they are expected to have acceptable knowledge, attitude, and practice with respect to correct toothbrush maintenance and use.

In this study, no statistically significant difference was found between the nursing and medical students in the total scores of knowledge and attitude. A study in India (2018) showed dental students had better knowledge and attitude about toothbrush maintenance and replacement [18]. Another study in Tanzania (2015) indicated the nursing students had acceptable knowledge about oral hygiene [19]. A study in India (2012) showed the nursing students had good knowledge and attitude about oral hygiene [12]. Another study in India (2012) indicated the nursing students had significantly higher knowledge and better attitude regarding oral health than nurses [20].

Moreover, a study in Kuwait (2003) revealed the medical students had little knowledge about oral health [14]. A study in Hong Kong (2015) reported the nursing students had little knowledge about oral hygiene [15]. The results of the current research were in line with those of some of the above-mentioned studies, indicating that the medical and nursing students had insufficient knowledge and unfavorable attitude with regard to toothbrush maintenance and use. The medical and nursing students, as the future healthcare providers of the society, need to have adequate knowledge and a favorable attitude about oral hygiene.

The medical and nursing students had similar and average practice in toothbrush maintenance and use in all items. The nursing students had better practice in using the toothbrush cap so that most of them used the cap after the toothbrush dried, but most medical students used the cap before the toothbrush dried. The results of a study in Kuwait (2003) and a study in Hong Kong (2015) showed the nursing students had unacceptable practice in oral hygiene [14, 15]. The findings of the present study confirmed the results of the above studies with respect to the poor practice of students in oral hygiene. On the other hand, the studies conducted in India (2012) and Tanzania (2015) reported the nursing students had acceptable practice in oral hygiene [12, 19], which is in contrast with the findings of the present study. In the above studies, the nursing students had acceptable knowledge and a favorable attitude, so it is not unexpected that they have a favorable practice. However, the nursing and medical students in the present research had unacceptable knowledge and attitude about toothbrush maintenance and use, which normally affected their practice.

In the present study, most nursing and medical students washed their toothbrushes before and after tooth brushing. Washing the toothbrush before and after tooth brushing is necessary because the toothbrush contains a significant number of microorganisms after tooth brushing [21]. On the other hand, a wet toothbrush can increase bacterial growth and lead to accumulation of microbes in toothbrush [22]. Therefore, it is important to dry the toothbrush before using the cap. In the current study, most nursing and medical students kept their toothbrush out of water closet. Keeping the toothbrush in the bathroom can cause toothbrush contamination and oral infections [6, 7]. In addition, more than half of the students in the present study replaced their toothbrushes every 2-3 months, which seems to be an appropriate activity. A toothbrush should be replaced every three months [23].

Since oral health is very important and the medical and nursing students had good practice in this regard, it seems necessary to provide them with more training. The use of internet-based methods can be an effective strategy to promote the knowledge, attitude, and practice of nursing and medical students [24].

The present study had several limitations. Since this study was cross-sectional, it was not possible to determine a causal relationship between students' major and their knowledge, attitude, and practice. Moreover, data were collected through self-report, which might have affected the accuracy of the results. However, the researchers made an attempt to minimize this limitation by assuring the participants that the questionnaires would remain anonymous.

## 5. Conclusion

Doctors and nurses as healthcare providers are responsible for the health of patients. Therefore, they are expected to have acceptable knowledge, attitude, and practice regarding oral health principles. The medical and nursing students in the present research did not have favorable knowledge, attitude, and practice with respect to toothbrush maintenance and use. They had similar knowledge about all items of toothbrush maintenance and use. As for most items of toothbrush maintenance and use, the medical and nursing students had similar attitudes and practices. This study was carried out on the medical and nursing students. Future studies are recommended to be performed on other students, especially dental students. Further studies are also advised to explore the effect of educational interventions on the knowledge, attitude, and practice of medical students.

## Figures and Tables

**Table 1:** Demographic characteristics of medical and nursing students (*n* = 580).

Variables		Nursing students number (%)	Medical students number (%)	Test results
Sex	Male	118 (45.4)	170 (53.1)	*X* ^*2*^ = 3.43
	Female	142 (54.6)	150 (46.9)	NS^‡^

Age		Median (IQR^†^)	Median (IQR)	*Z* ^‡‡^ = −0.90
		21 (3)	21 (3)	NS

^‡^Nonsignificant; ^†^interquartile range; ^‡‡^based on Mann–Whitney *U* test.

**Table 2:** Comparison of attitude, knowledge, and practice scores of medical and nursing students about the maintenance and use of a toothbrush.

Variables	Nursing students		Medical students		Test results
	Median (IQR^†^)	Mean ± SD	Median (IQR)	Mean ± SD^††^	
Knowledge	60 (30)	54.53 ± 17.18	60 (30.00)	54.21 ± 17.42	*Z* ^‡^ = −0.27
					NS^‡‡^

Attitude	50 (33.33)	49.67 ± 19.17	50 (33.33)	51.87 ± 18.28	*Z* = −1.16
					NS

Practice	50 (30)	51.00 ± 19.50	50 (20.00)	49.87 ± 17.52	*Z* = −0.93
					NS

^†^Interquartile range; ^††^standard deviation; ^‡^based on Mann–Whitney *U* test;^‡‡^nonsignificant.

**Table 3:** Comparison of knowledge of nursing and medical students about toothbrush maintenance and use of a toothbrush.

Items		Nursing students number (%)	Medical students number (%)	Test results
What water temperature is suitable for washing the toothbrush?	Hot	40 (15.4)	46 (14.4)	*X* ^*2*^ = 0.40
	Cold	61 (23.5)	82 (25.6)	NS^†^
	Lukewarm	159 (61.2)	192 (60.0)	

What is the right time to use toothbrush cap?	Immediately after brushing	64 (24.6)	79 (24.7)	*X* ^*2*^ = 0.00
	After drying the toothbrush	196 (75.4)	241 (75.3)	NS

When should a toothbrush be replaced? (in months)	1-2	39 (15.0)	47 (14.7)	*X* ^*2*^ = 1.25
	2-3	142 (54.6)	162 (50.6)	NS
	6	79 (30.4)	111 (34.7)	

What is the best tooth brushing method?	Horizontal-rotary	56 (21.5)	79 (24.7)	*X* ^*2*^ = 2.19
	Vertical-rotary	176 (67.7)	198 (61.9)	NS
	Irregular	28 (10.8)	43 (13.4)	

What is the right place for keeping the toothbrush?	Inside the bathroom	55 (21.2)	55 (17.2)	*X* ^*2*^ = 1.46
	Out of the bathroom	205 (78.8)	265 (82.8)	NS

What is the right method for washing the toothbrush?	Complete washing with hot water	76 (29.2)	99 (30.9)	*X* ^*2*^ = 6.24
	Complete washing with cold water	99 (38.1)	133 (41.6)	NS
	Rinsing with hot water	32 (12.3)	47 (14.7)	
	Rinsing with cold water	53 (20.4)	41 (12.8)	

When should a toothbrush be washed?	Before brushing	30 (11.5)	22 (6.9)	*X* ^*2*^ = 4.52
	After brushing	64 (24.6)	93 (29.1)	NS
	Before and after brushing	166 (63.8)	205 (64.1)	

How long should tooth brushing last?	1-2 minutes	24 (9.2)	43 (13.4)	*X* ^*2*^ = 4.47
	2-3 minutes	143 (55)	152 (47.5)	NS
	More than 3 minutes	82 (31.5)	113 (35.3)	
	Less than 1 minute	11 (4.2)	12 (3.8)	

What is the right method for keeping toothbrush after brushing?	Hanging	197 (75.8)	254 (79.4)	*X* ^*2*^ = 1.07
	Keeping in water upside down	63 (24.2)	66 (20.6)	NS

^†^Nonsignificant.

**Table 4:** Comparison of nursing and medical students' attitudes toward toothbrush maintenance and use.

Items		Nursing students number (%)	Medical students number (%)	Test results
Toothbrush material affects its lifespan	I agree	238 (91.5)	271 (84.7)	*X* ^*2*^ = 6.26
	I disagree	22 (8.5)	49 (15.3)	*P*=0.01

Bathroom is a proper place for keeping the toothbrush	I agree	54 (20.8)	56 (17.5)	*X* ^*2*^ = 0.99
	I disagree	206 (79.2)	264 (82.5)	NS^†^

I can brush my teeth more than the recommended time if I do not brush my teeth harshly.	I agree	121 (46.5)	169 (52.8)	*X* ^*2*^ = 2.25
	I disagree	139 (53.5)	151 (47.2)	NS

Rinsing is sufficient to reduce toothbrush contamination	I agree	129 (49.6)	139 (43.4)	*X* ^*2*^ = 2.20
	I disagree	131 (50.4)	181 (43.4)	NS

The harder the toothbrush is, the better its material is	I agree	51 (50.4)	45 (14.1)	*X* ^*2*^ = 3.20
	I disagree	209 (80.4)	275 (85.9)	NS

Toothbrushes made in foreign countries are more durable	I agree	192 (73.8)	244 (76.3)	*X* ^*2*^ = 0.44
	I disagree	68 (26.2)	76 (23.8)	NS

^†^Nonsignificant.

**Table 5:** Comparison of nursing and medical students' practice toward toothbrush maintenance and use.

Items		Nursing students number (%)	Medicine students number (%)	Test results
How often do you brush your teeth a day?	Once	26 (10.0)	48 (15.0)	*X* ^*2*^ = 3.71
	Twice	96 (36.9)	120 (37.5)	NS^†^
	Thrice	138 (53.1)	152 (47.5)	

What type of toothbrush do you use?	Soft	78 (30.0)	89 (27.8)	*X* ^*2*^ = 0.42
	Medium	164 (63.1)	206 (64.4)	NS
	Hard	18 (6.9)	25 (7.8)	

What tooth brushing method do you use?	Horizontal-rotary	71 (27.3)	77 (24.1)	*X* ^*2*^ = 2.69
	Vertical-rotary	155 (59.6)	186 (58.1)	NS
	Irregular	34(13.1)	57 (17.8)	

How often do you change your toothbrush?(in months)	2-3	127 (48.8)	142 (44.4)	*X* ^*2*^ = 2.11
	4-5	93 (35.8)	115 (35.9)	NS
	Whenever the toothbrush hair is worn or bent	40 (15.4)	63 (19.7)	

Where do you keep your toothbrush?	Inside the bathroom	81 (31.2)	94 (29.4)	*X* ^*2*^ = 0.21
	Out of the bathroom	179 (68.8)	226 (70.6)	NS

How do you wash your toothbrush?	Rinsing with hot water	64 (24.6)	84 (26.3)	*X* ^*2*^ = 3.78
	Complete washing with hot water	42 (16.2)	56 (17.5)	NS
	Rinsing with cold water	61 (23.5)	89 (27.8)	
	Complete washing with cold water	93 (35.8)	91 (28.4)	

When do you wash your toothbrush?	Before brushing	29 (11.2)	25 (7.8)	*X* ^*2*^ = 2.86
	After brushing	95 (36.5)	134(41.9)	NS
	Before and after brushing	136 (52.3)	161(50.3)	

How do you keep your toothbrush after brushing?	Hanging	200 (76.9)	259 (80.9)	*X* ^*2*^ = 1.40
	Keeping in water upside down	60 (23.1)	61 (19.1)	NS

How long do you brush your teeth? (in minutes)	≤1	21 (8.1)	34 (10.6)	*X* ^*2*^ = 1.27
	1-2	88 (33.8)	102 (31.9)	NS
	2-3	84 (32.3)	99 (30.9)	
	≥3	67 (25.8)	85 (26.6)	

Do you dry your toothbrush after brushing?	Yes	104 (40)	119 (37.2)	*X* ^*2*^ = 0.47
	No	156 (60)	201 (62.8)	NS

If you use a toothbrush cap, when do you use it?	After drying the toothbrush	152 (58.5)	156 (48.8)	*X* ^*2*^ = 5.43
	Immediately after washing the toothbrush	108 (41.5)	164 (51.2)	*P*=0.020

^†^Nonsignificant.

## Data Availability

The identified datasets analyzed during the current study are available from the corresponding author on reasonable request.
